# Perceived Digital Value Toward Continuous Intention to Use Among Mobile Payment Users During Pandemic Outbreak

**DOI:** 10.3389/fpsyg.2022.892821

**Published:** 2022-05-13

**Authors:** Ratyuhono Linggarnusantra Putra, Margono Setiawan, Ananda Sabil Hussein, Agung Yuniarinto

**Affiliations:** Department of Management, Faculty of Economics and Business, University of Brawijaya, Malang, Indonesia

**Keywords:** customer value, perceived digital value, mobile payment, demography, COVID−19

## Introduction

Mobile payment (M-Payment) is a payment method using an application in a mobile device, such as mobile phones, gadgets, etc. It has a deposit feature where the users can put their money and use it in many types of transactions. The users can use it for cashless payment while buying goods or services, transferring money to other accounts, paying utility bills, etc. M-Payment offers flexibility where the users can perform their transaction anywhere and anytime (Dahlberg et al., 2008; Thakur and Srivastava, 2014; Tam and Oliveira, 2017; Fahlevi and Alharbi, 2021). M-Payment in Indonesia recently has become popular. PricewaterhouseCoopers' survey about Global Consumer Insight 2019 indicated that there was an increment on the active user number from 38% in 2018, becoming 47% in 2019 during the pandemic outbreak. Aligned with the increasing number of users, the number of M-Payment providers has also increased. Data from katadata.co.id showed there were 37 M-Payment providers, which were 21 providers from financial technology companies, 11 providers from banks, and 5 providers from telecommunication companies in the end of May 2019. In 2020, there were 47 companies of M-Payment providers that are officially registered in the Bank of Indonesia both from private sectors and governmental institutions. The number of M-Payment providers creates a competitive competition. Each provider is expected to be able to get new users or at least maintain its existing users. Data from iPrice in 2019 indicated that the market share based on number of application download and active users is fluctuating, even though there is a provider that consistently stays in the first rank. This situation becomes a challenge for majority of the providers to ensure business sustainability. Thus, continuance intention to use the M-Payment among the existing users is important to establish business sustainability when expanding their market share is more challenging and difficult.

Continuance intention to use is another meaning of loyalty that has been a significant element for user retention (Zhao and Kurnia, 2011). Oliver (2010) explained that customer loyalty can increase the company's profit since the company does not need to spend much money for attracting new customers. This is relevant to the phenomenon that M-Payment providers at least need to ensure their users to use their brand continuously to establish business sustainability. Several previous studies have discussed the antecedents of continuance intention to use such as perceived value (Chiu et al., 2014), satisfaction (Kim et al., 2009; Kuo et al., 2009; Zhou, 2013), and various dimensions of perceived value, such as functional value, emotional value, social value, and monetary value (Wang et al., 2004; Deng et al., 2010; Kim et al., 2010).

In accordance with the previous studies, most of the studies found significant impact from perceived value to continuance intention, even though some gaps still exist. The first gap is about the perceived value's dimensions. Sabiote-Ortiz et al. (2016) explained that perceived value can be described as a subjective construct that will be different between consumers, culture, and over time. This means that the perceived value's dimensions are different in each research setting. Related to digital or high-tech product, Abaidi and Vernette (2017) mentioned that only few studies investigate the impact of digitalization on perceived value from a holistic perspective. Several previous studies have shown that there are some differences in terms of perceived value of digitized or intangible products compared to tangible or physical products. For example, the changes offer consumers new benefits, such as everyone can access it anytime and also enhance its convenience and usefulness (Wolfinbarger and Gilly, 2001; Chain and Granitz, 2012; Fahlevi, 2021). The product digitalization also affects consumer's emotional bond and its feeling to the product because the sensory dimension is reduced or even not there (Bogart, 1992; Bouwman and Van de Wijngaert, 2002; Flavián and Gurrea, 2006; Siddiqui and Turley, 2006; Chain and Granitz, 2012). In terms of monetary, Rushton and Carson (1989) described that intangible product caused more complicated monetary evaluation, for example, overall cost needs to consider learning time that is time-consuming for certain consumers (Rowley, 2008). Docters et al. (2011) in their study on “pricing in the digital world” explained the pricing concept in digitized product is context driven, not feature driven. Related to social value, Lemon (2016) mentioned that digital technology can change a consumer's expectation and behavior because the boundaries between humans and machines are blurred, even though it can still provide interactive personalization through information exchange between organizations and consumers (Parise et al., 2016). By considering the value changes on intangible or digitized product and to add novelty, this study developed the idea of perceived digital value (PDV) to replace the construct of perceived value. PDV is more contextual than the general concept of perceived value. It is expected to become a new concept that is more applicable for future studies on digital or high-tech products. To fill in this gap and establish the PDV concept, this study explored the PDV's dimensions in the context of continuance intention to use M-Payment.

The second gap is about the demographic profile that affects the significance level of perceived value to continuance intention. Ryu (2017) in his study about fintech found different significance levels when tested all respondents without a demography category and when tested with categorized respondents as early adopters and late adopters. The result that was significant in all the respondents changed to be not significant in the early adopters category, when the respondents were categorized. Through the findings, the study concluded that different expectations and interests between consumers may result in different significance levels. However, there has been no study capturing demography as a moderating variable that can affect the significance level between perceived value to continuance intention, especially in the context of M-Payment usage. Integrating demography as a moderating variable will improve the predictive power of the research model and generate comprehensive results that can capture significance level differences between demography categories.

Based on the research gaps, this study has two research objectives: The first objective is to explore the dimensions of PDV, and the second objective is to scrutinize the role of demography as a moderating variable between PDV and continuance intention to use. Upon the completion, this study provided both theoretical and practical contributions. For the theoretical contribution, this study established the concept of PDV and explored its dimensions from the perspective of M-Payment users in Indonesia, especially Jakarta, including its relationship with continuance intention to use. For the practical contributions, this study provided insight for M-Payment providers to formulate segmented strategies to ensure continuance intention to use among their users.

## Consumption Value Theory

Consumption value theory is a famous conceptual theory related to perceived value that is often used to predict consumer behavior. It has been examined as an antecedent of various behavior outputs, such as subscription intention, customer satisfaction, and customer loyalty (Chen and Dubinsky, 2003). In the field of service marketing, perceived value has recently been a concern of researchers due to the increasing number of consumers who attach the importance of value (El-Adly and Eid, 2015). In order to understand more deeply the perceived value of the user, there were several studies that had looked at the perceived value in a multidimensional perspective.

For developing the theory to fit for digitized product or service, the author should consider the phenomenon of digital or intangible products or services, and look for the potential of value differences between intangible and tangible products. This study used the existing perceived value concept, which is PERVAL scale by Sweeney and Soutar (2001) to be developed further to establish the PDVconcept in a research model to increase the relevancy of consumption value theory with digitized products or services. Furthermore, this new model also considered demography as a moderating variable between PDV as an independent variable and continuance intention to use as a dependent variable. Demography as a moderating variable was used to capture the potential of significance level differences among demography categories of the respondents.

## Methods

**Figure 1:**
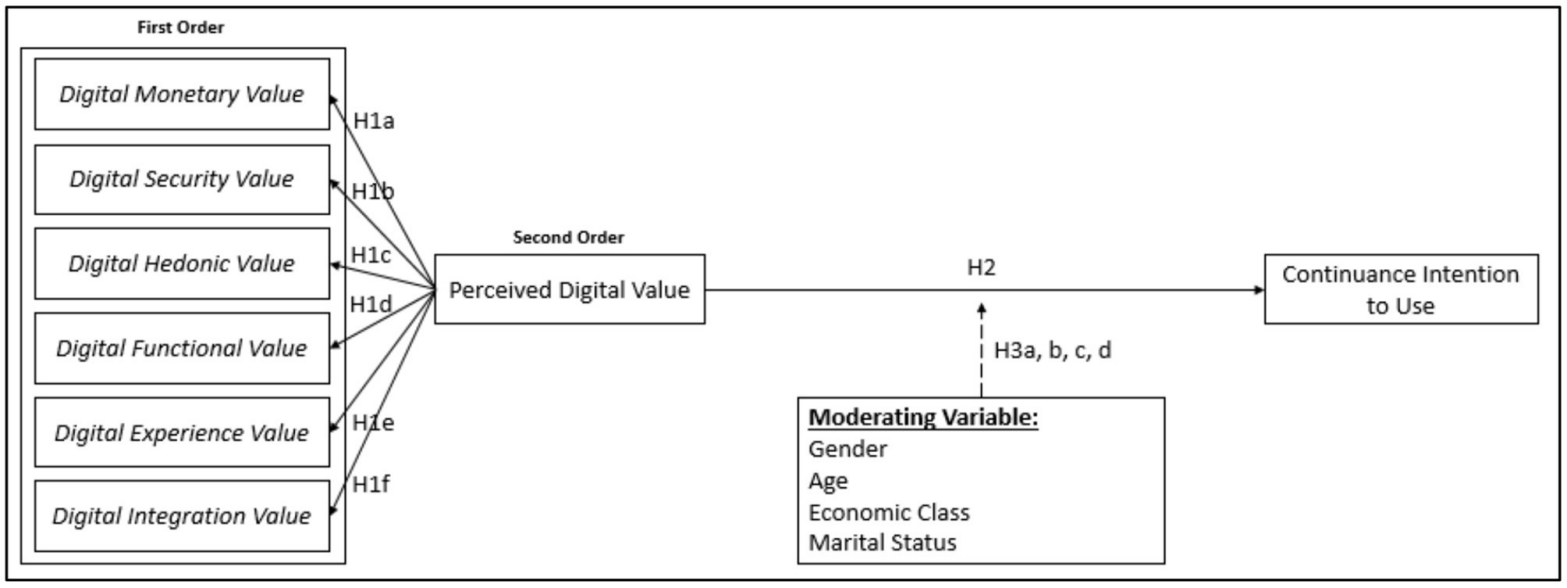
Conceptual model.

In order to answer the two objectives, then a new conceptual model can be designed which can be seen in Figure 1. This study conducted two consecutive smaller studies: The first study is categorized as an exploratory study that aims to explore the dimensions of PDV. In exploring the dimensions of PDV, this study followed the steps proposed by Churchill (1979). The results of the first study were used in the second study, which was an explanatory study to test the hypotheses. To answer the first research objective, an exploratory study was conducted through three series of focus group discussions (FGDs) and two surveys *via* a self-administered questionnaire. Using a convenience sampling approach, each FGD session was done around 90 min. Every session consisted of six M-Payment users and was led by a moderator who was accompanied by a note taker. The participants must have an adequate frequency in using M-Payment, which is minimum 2 times in a week, and they must use the M-Payment willingly. The participants must also spend most of their time in the Jakarta area. For the survey, the respondent's criteria are the same as the FGD participants.

## Discussion

After exploring the dimensions of PDV, the second research objective is to determine the effect of PDV on continuance intention to use. The data analysis results showed that PDV has a positive significant effect on continuance intention to use, which means Hypothesis 2 is accepted. The finding proves that the PDV is considered to be a crucial factor affecting an important marketing construct that results in positive continuance intention to use. In addition, this study also explored the effect of some demography categories as moderating variables between PDV and continuance intention to use. Based on the result of a multi-group analysis, gender, age, and economic class were found to have a significant effect as a moderating variable between PDV and continuance intention to use, whereas marital status was found not significant. The result showed that there are differences in the impact of PDV on continuance intention to use between groups in gender, age, and economic class. Male in the gender category, adult in the age category, and upper in the economic class category are groups that have the highest t-statistic score in their demography category. It means users in those groups received PDV as a meaningful factor that can impact their continuance intention to use M-Payment compared to other groups in their demography category.

After completing the proposed research objectives, both theoretical and practical contributions have been provided by this study. From a theoretical standpoint, this study explored the dimensions of PDV. The exploratory study suggested that digital monetary value, digital security value, digital hedonic value, digital functional value, digital experience value, and digital integration value are the dimensions of PDV. Apart from contributing to the provider company of M-Payment and marketing literature by discovering these dimensions, this study provides a comprehensive conceptual model, explaining the relationship between PDV and continuance intention to use in the context of M-Payment users in Jakarta, Indonesia. The next theoretical contribution is about the moderating role of some demography categories, such as gender, age, economic class, and marital status in the relationship between PDV and continuance intention to use. This study found a significant moderating effect from gender, age, and economic class, whereas marital status was not significant.

## Conclusion

This study provides new insight into marketing academics and practitioners, indicating that digital monetary value, digital security value, digital hedonic value, digital functional value, digital experience value, and digital integration value are proven to be important constructs as the dimensions of PDV in enhancing continuance intention to use M-Payment. These findings simply state that M-Payment providers should ensure that their M-Payment brand provides value for their users that will enhance continuance intention to use. The users will thus perceive high value and have a higher level of intention to use the M-Payment continuously. Furthermore, those constructs will improve the user intention to use M-Payment continuously.

## Author Contributions

RP contributed to conceptualization, methodology, investigation, curation, analysis, funding acquisition, and writing. AH helped in investigation, curation, analysis, and writing. MS and AY helped in review, analysis, and writing. All authors contributed to the article and approved the submitted version.

## Conflict of Interest

The authors declare that the research was conducted in the absence of any commercial or financial relationships that could be construed as a potential conflict of interest.

## Publisher's Note

All claims expressed in this article are solely those of the authors and do not necessarily represent those of their affiliated organizations, or those of the publisher, the editors and the reviewers. Any product that may be evaluated in this article, or claim that may be made by its manufacturer, is not guaranteed or endorsed by the publisher.
